# Chest Pain Caused by Thoracic Spine Plasmacytoma: A Case Report

**DOI:** 10.7759/cureus.38131

**Published:** 2023-04-25

**Authors:** Carlos Gaibor, Nighat Qadri

**Affiliations:** 1 Internal Medicine, St. Luke's Hospital, Chesterfield, USA

**Keywords:** lexiscan, plastocytoma, thoracic mass, multiple myeloma, chest pain

## Abstract

We present a case of a thoracic mass causing chest pain that was initially attributed to coronary artery disease due to his comorbidities. However, during the Lexiscan stress test, a thoracic spinal mass was incidentally found. This case showed the importance of being cognizant of other causes of chest pain and a rare presentation of multiple myeloma.

## Introduction

Multiple myeloma (MM) is a malignant disorder involving plasma cells in which abnormal immunoglobulin is overproduced [1]. MM is an uncommon cancer, accounting for 1 to 2% among all cancers and 17% among all hematologic malignancies [2], and is often diagnosed between 65 and 74 years old [3]. Its clinical presentation includes anemia, bone pain, elevated creatinine, fatigue, generalized weakness, hypercalcemia, and weight loss [3]. MM could be subclassified based on the type of immunoglobulins, with the most common being immunoglobulin G (IgG) with 52%, immunoglobulin A (IgA) with 21%, and Kappa and lambda light chains with 16% [3]. MM could be diagnosed when a bone marrow biopsy showed more than 60% of clonal bone marrow plasma cells, but when more than 10% of clonal bone marrow plasma cells are found, MM could only be diagnosed when it is associated with organ damage or a laboratory marker suggestive of MM [4]. Similarly, immunohistochemical staining, immunofluorescent studies, and flow cytometry could assist in the identification of the immunophenotype, and fluorescence in situ hybridization (FISH) is used for detecting cytogenetic abnormalities [5]. Regarding prognosis, if MM is left untreated, the median survival is approximately one year [6], so high-dose chemotherapy followed by autologous stem cell transplant (ASCT) increases the median overall survival to more than five years [7]. 

## Case presentation

**Figure 1:**
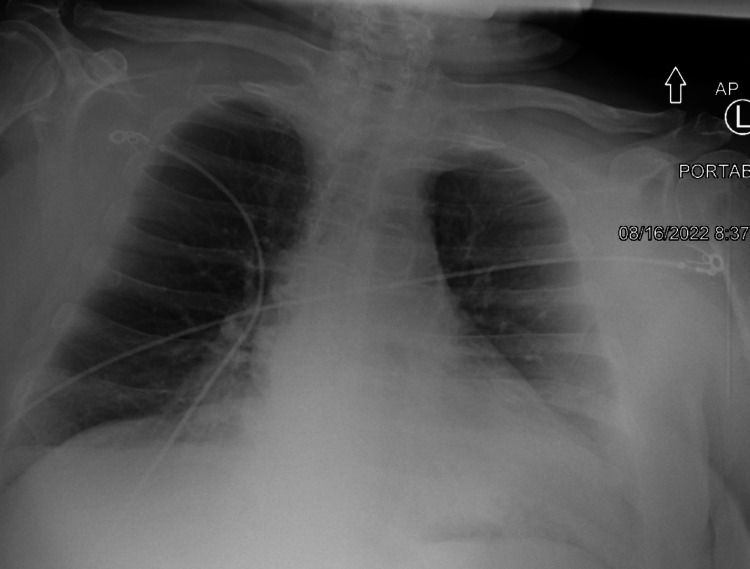
CXR showing borderline cardiomegaly. CXR: chest x-ray.

The patient is a 72-year-old white male with a significant past medical history of obesity, diabetes, hypothyroidism, hypertension, hyperlipidemia, coronary artery disease, chronic kidney disease, unspecified chronic anemia, and obstructive sleep apnea who presents with chest pain. The chest pain is midline, pressure-like, four out of 10 in severity, nonradiated and aggravated with movement of the arms upwards. The patient mentions having experienced mild chest pain associated with exertional shortness of breath with just a block for more than a year; however, the chest pain has exacerbated since yesterday. Moreover, the patient denies fever, unintentional weight loss, headache, diaphoresis, nausea, vomiting, and abdominal pain. Laboratories were relevant for a white blood cell count of 7.2 k/uL, hemoglobin of 11.1 g/dl, blood urea nitrogen (BUN) of 24 mg/dl, creatinine of 1.3 mg/dl, and troponin was negative twice (Table 1); electrocardiogram was negative for ischemic changes; and chest x-ray showed borderline cardiomegaly (Figure 1). 

**Table 1:** Initial laboratory.

Laboratory	Result	Normal range
White blood cells	7.2 K/uL	4.3-10.0 K/uL
Hemoglobin	11.1 g/dl	13.6-16.5 g/dl
Hematocrit	34.60%	40.0-48.0%
Mean corpuscular volume (MCV)	87.4 fL	82.0-99.0 fL
Mean corpuscular hemoglobin (MCH)	28.0 pg	27.2-32.6 pg
Mean corpuscular hemoglobin concentration (MCHC)	32.1 g/dl	31.5-35.5 g/dl
Red cell distribution width (RDW)	14.50%	11.5-14.5%
Sodium	139 mmol/L	137-145 mmol/L
Potassium	4.6 mmol/L	3.5-4.9 mmol/L
Chloride	109 mmol/L	98-107 mmol/L
Bicarbonate	20 mmol/L	22-30 mmol/L
Blood urea nitrogen (BUN)	24 mg/dl	9-20 mg/dl
Creatinine	1.3 mg/dl	0.7-1.3 mg/dl
Glucose	181 mg/dl	74-106 mg/dl
Calcium	9.2 mg/dl	8.4-10.2 mg/dl
Protein total	6.6 g/dl	6.5-8.6 g/dl
Albumin	3.4 g/dl	3.5-5.0 g/dl
Alkaline phosphatase	89 U/L	38-126 U/L
Bilirubin, total	0.2 mg/dl	0.2-1.3 mg/dl
Aspartate aminotransferase (AST)	27 U/L	14-54 U/L
Alanine transaminase (ALT)	25 U/L	≤50 U/L

**Figure 2:**
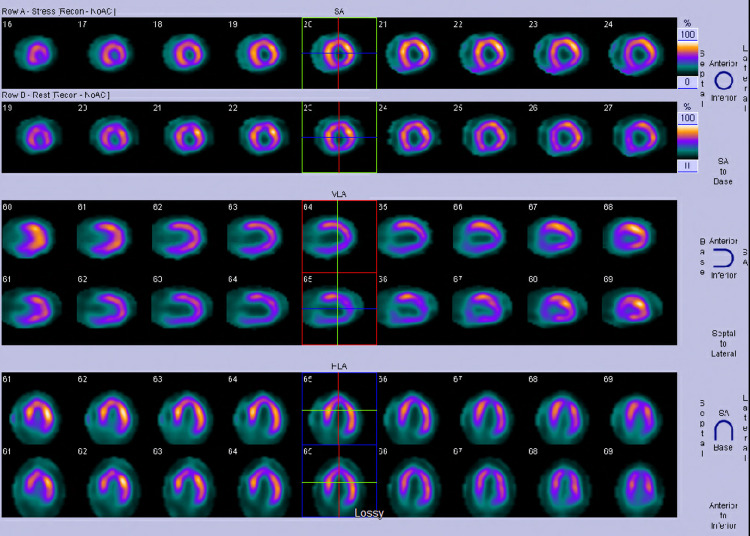
Lexiscan stress myocardial perfusion imaging (MPI) is clinically and electrocardiographically negative for ischemia.

**Figure 3:**
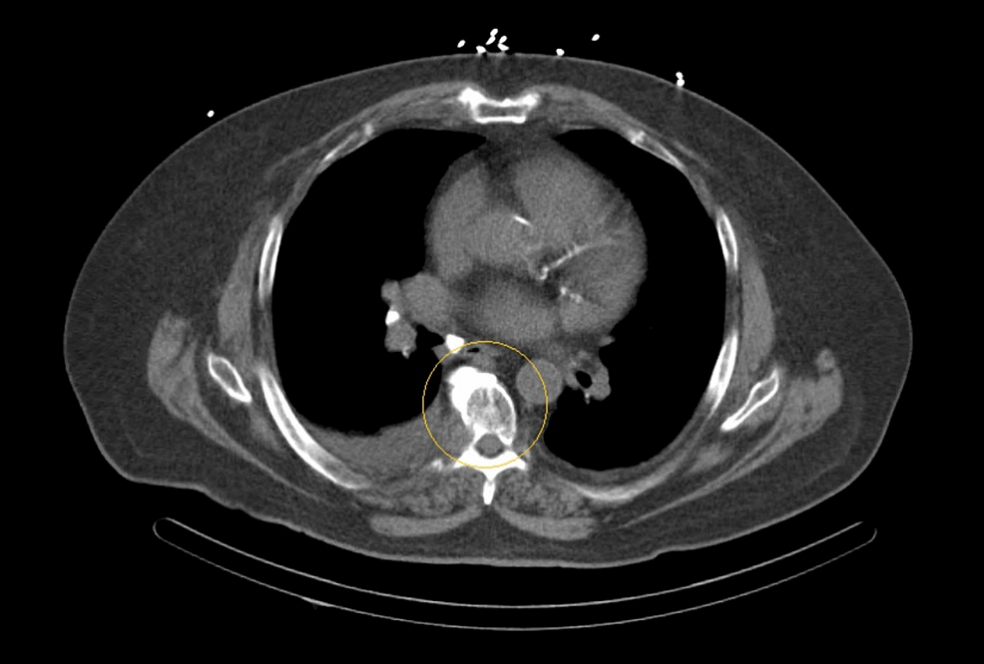
Thoracic mass revealed on the limited computerized tomography (CT) imaging of the chest.

Based on a high suspicion of coronary artery disease, cardiology was consulted and recommended Lexiscan stress myocardial perfusion imaging (MPI) (Figure 2), which was clinically and electrocardiographically negative for ischemia with a normal myocardial perfusion and a normal ejection fraction of 60%. Nonetheless, a thoracic mass was reported during limited computerized tomography (CT) imaging of the chest that is performed for the purposes of attenuation correction as part of a nuclear medicine myocardial perfusion scan (Figure 3). 

**Figure 4:**
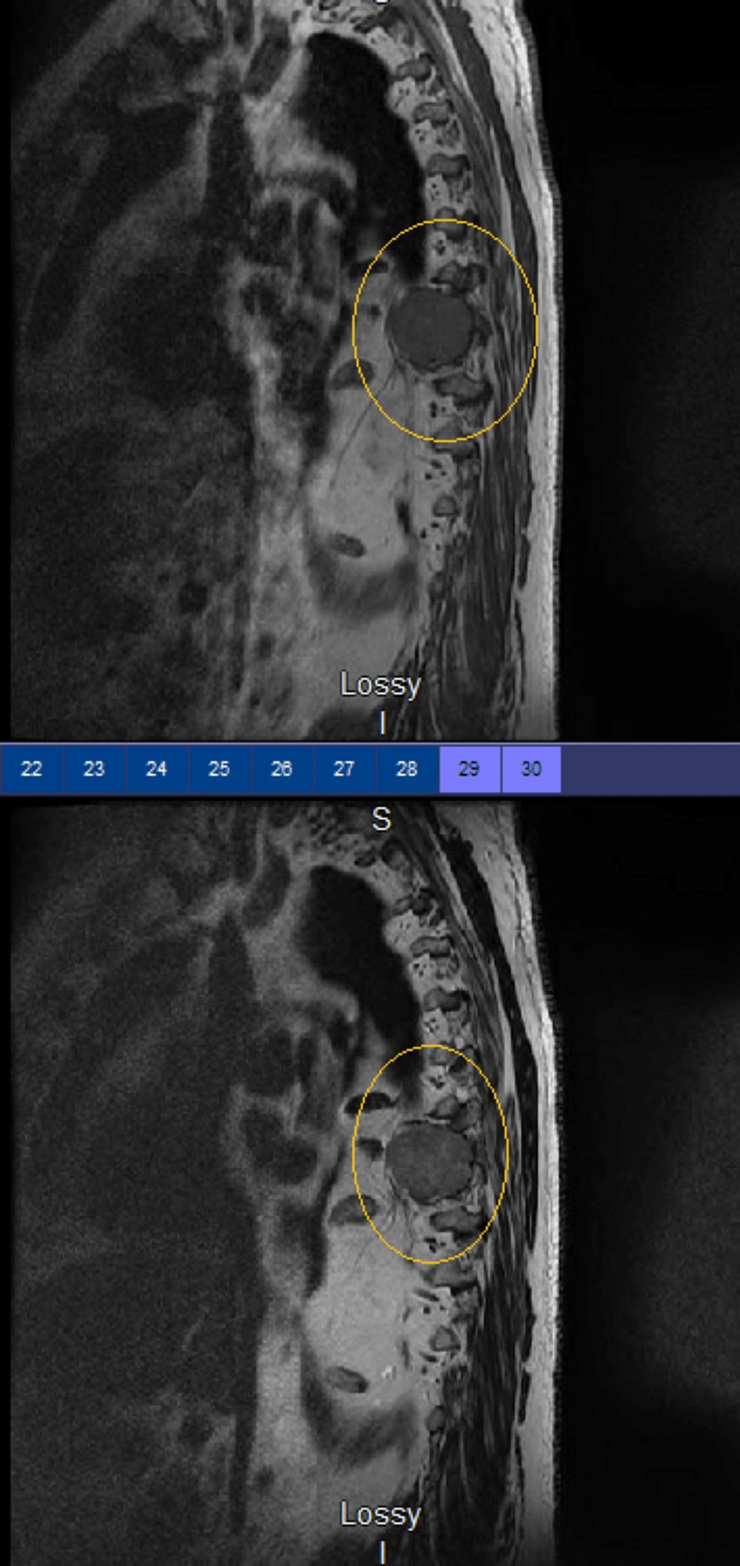
MRI of the thoracic spine with and without contrast revealing a T8-centered infiltrative solid destructive mass lesion with mild spinal cord compression.

As a result, a thoracic magnetic resonance imaging (MRI) with and without was ordered and revealed a thoracic (T) 8-centered, restrictive, solid, destructive mass lesion that compressed the spinal cord (Figure 4). 

**Figure 5:**
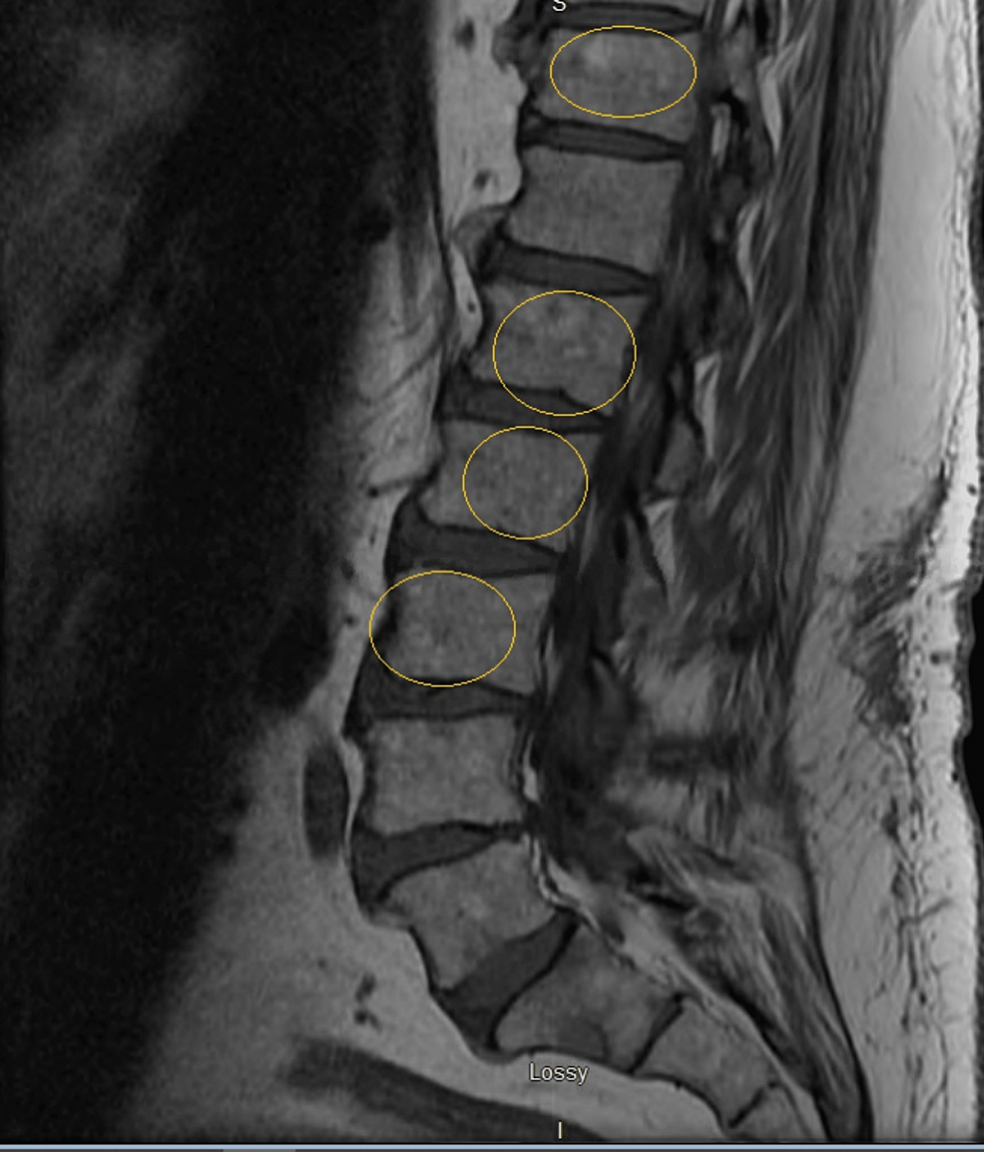
MRI of the lumbar spine revealing multiple enhancements in L1 (lumbar) and S1 (sacral) regions, indicative of metastasis.

**Figure 6:**
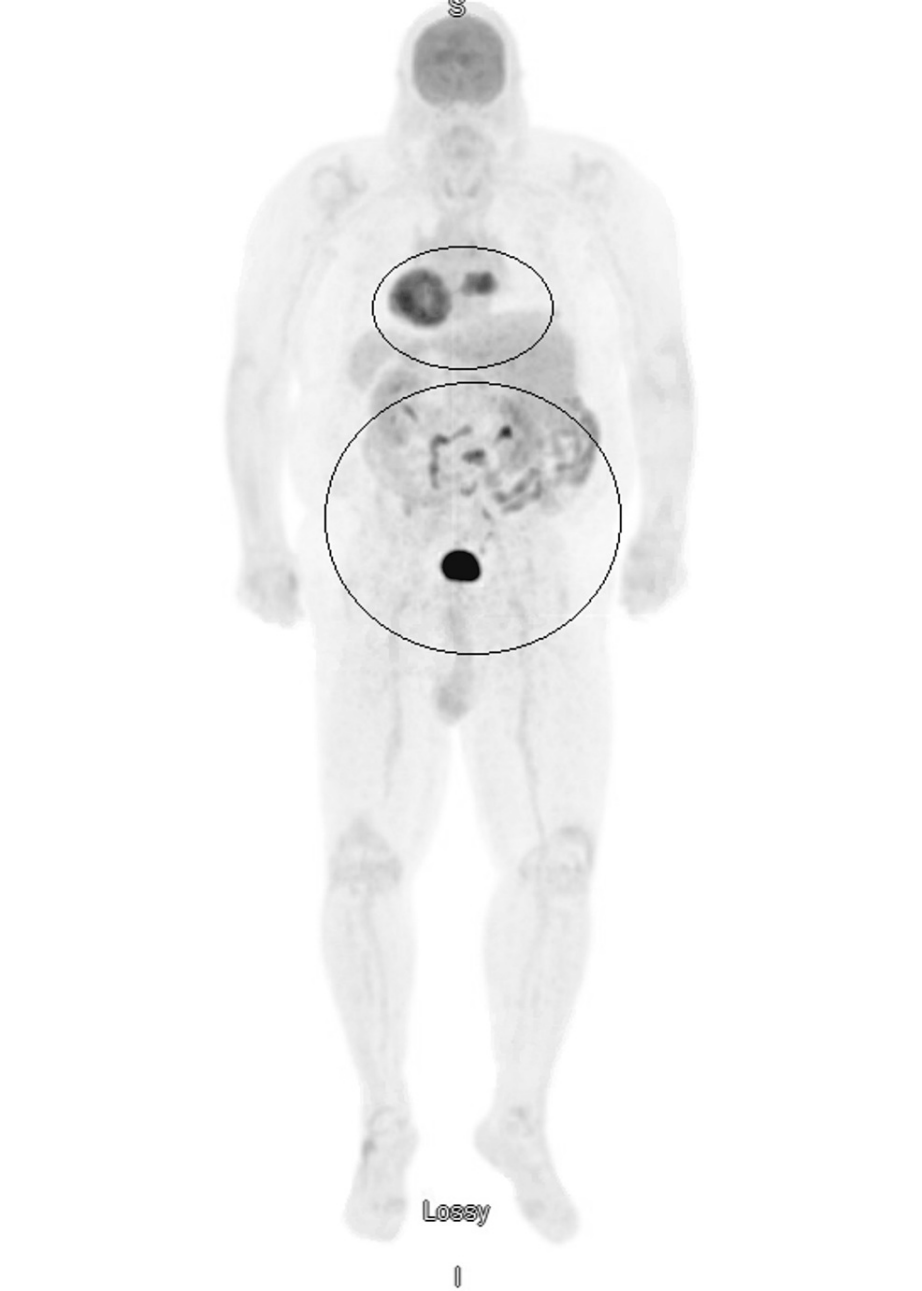
PET scan of the whole body showing a right paraspinal soft tissue mass. PET: positron emission tomography.

**Figure 7:**
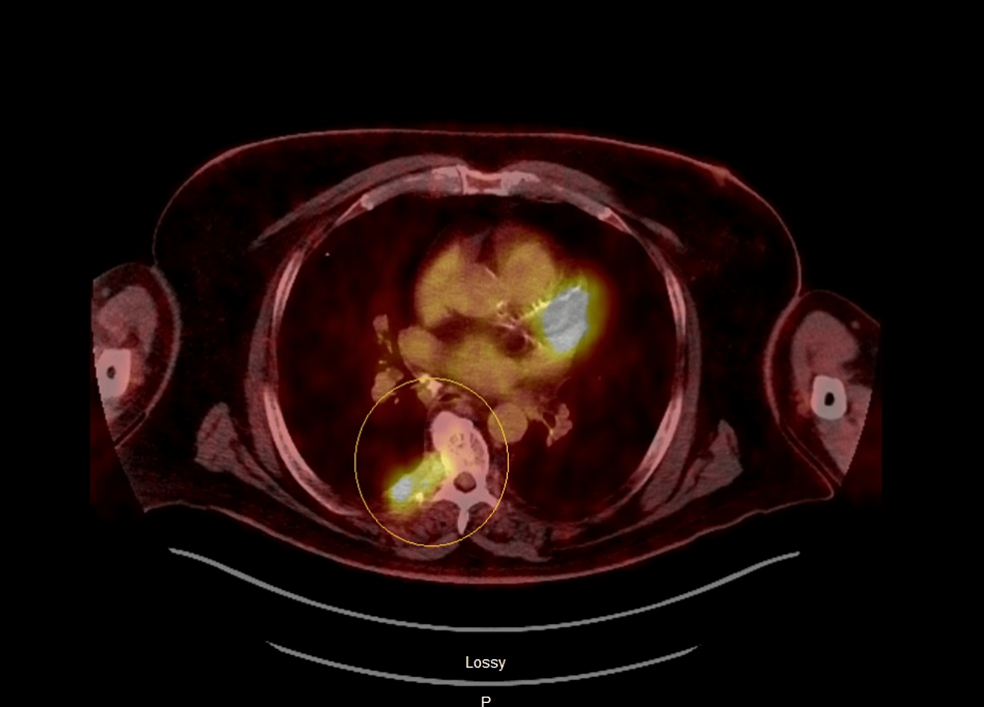
PET scan showing a right paraspinal soft tissue mass with PET destruction of the posterior eighth rib and adjacent vertebral body invading the spinal canal with all other smaller bony lesions, including the anterior right seventh rib and sternum. PET: positron emission tomography.

Therefore, neurosurgery and hematology-oncology were consulted for further management. Subsequently, an MRI of the lumbar spine revealed multiple enhancements in L1 (lumbar) and S1 (sacral) regions, indicative of metastasis (Figure 5). Thus, a positron emission tomography (PET) scan of the whole body (Figures 6, 7) was performed, showing a right paraspinal soft tissue mass with PET destruction of the posterior eighth rib and adjacent vertebral body invading the spinal canal with all other smaller bony lesions, including anterior right seventh rib, sternum, and lumbar spine. 

**Figure 8:**
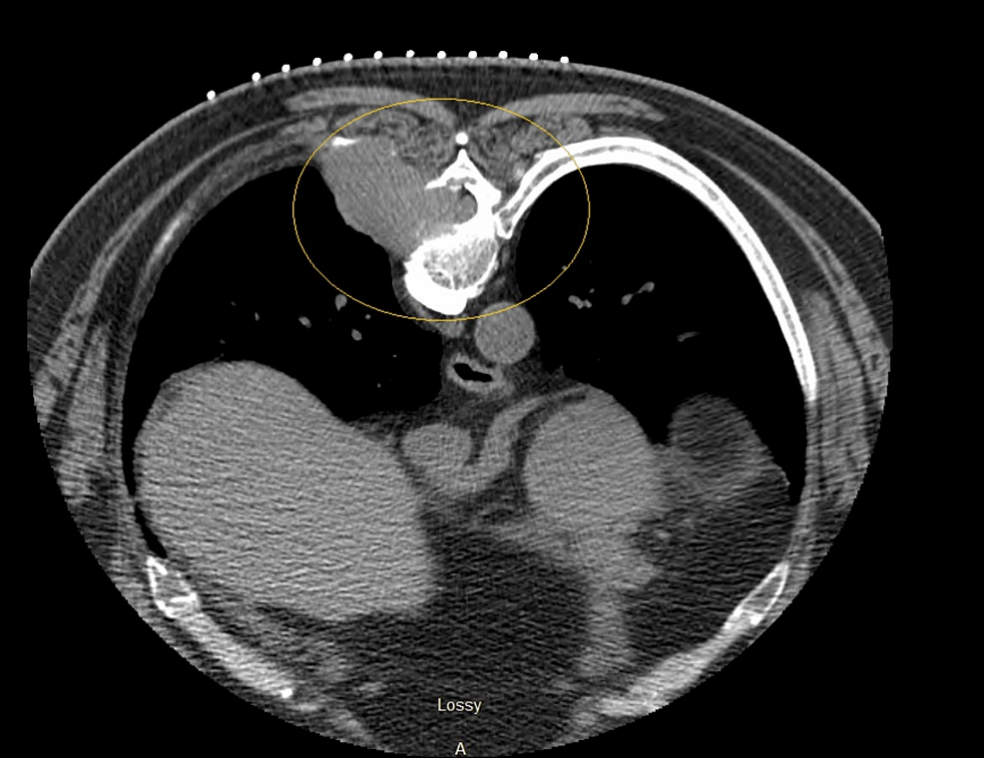
CT-guided core needle biopsy of the 5.6×3.5×3.2 cm mass lesion of the T8 vertebral body. CT: computed tomography.

Consequently, a computed tomography (CT)-guided core needle biopsy of the 5.6×3.5×3.2 cm mass lesion of the T8 vertebral body (Figure 8) alongside immunofixation and fluorescence in situ hybridization (FISH) were performed (Tables 2, 3, 4). So immunoglobulin G (IgG) kappa multiple myeloma with 10% plasma cells in the bone marrow with a standard risk (FISH revealed hyperdiploid cells) was diagnosed, for which the patient was managed with radiation therapy followed by chemotherapy with bortezomib, lenalidomide, and dexamethasone (VRd) with later consideration for hematopoietic cell transplantation (HCT).

**Table 2:** Immunoglobulin quantification.

	Value	Normal range
Immunoglobulin G (IgG)	346 mg/dl	603-1613 mg/dl
Immunoglobulin A (IgA)	186 mg/dl	61-437 mg/dl
Immunoglobulin M (IgM)	10 mg/dl	15-143 mg/dl

**Table 3:** Kappa/lambda light chains free with ratio.

Kappa light chain free	417.4 mg/L	3.3-19.4 mg/L
Lambda light chain free	20.5 mg/L	5.7-26.3 mg/L
Kappa/lambda ratio free	20.36	0.26-1.65

**Table 4:** Serum protein electrophoresis (SPEP).

Serum protein electrophoresis	Value	Normal range
Albumin fraction	2.89 g/dL	3.77-5.94 dl/L
Alpha 1 globulin	0.20 g/dL	0.06-0.26 g/dL
Alpha 2 globulin elevated	1.32 g/dL	0.47-1.03 g/dL
Beta globulin	0.71 g/dL	0.47-1.06 g/dL
Gamma globulin	0.37 g/dL	0.50-1.30 g/dL
Aminomethyltransferase monoclonal protein	0.66 g/dL	

## Discussion

This case presents an interesting case of a 72-year-old male with chest pain. Due to his significant cardiovascular risk factors, a lexiscan stress test was initially performed, but it was negative for ischemia. Nonetheless, it revealed a thoracic mass that was diagnosed as multiple myeloma, which usually presents with elevated calcium, renal failure, anemia, and bone lytic lesions [8]. In this case, multiple myeloma was not initially considered because of its atypical presentation, including normal serum calcium. Although mild elevated creatinine and mild anemia were found, these findings are nonspecific and could be related to a history of diabetes, which is a well-known cause of renal impairment. In addition, because the initial ischemic workup was negative, including a negative troponin and an unremarkable electrocardiogram, this patient could have been discharged or more conventional stress tests, such as an electrocardiogram stress test or even an echocardiogram stress test, could have been considered that were unable to detect this thoracic mass. Therefore, this diagnosis could have been missed or delayed, and his chest pain could have been attributed to more common causes such as gastroesophageal reflux. Therefore, this case showed the importance of being aware of other causes of chest pain, such as multiple myeloma causing chest pain [9], especially taking into consideration risk factors such as his age and perhaps raising the question of the importance of a lateral chest x-ray when other more conventional stress tests are used, such as those indicated above.

## Conclusions

This patient is a 72-year-old male with significant cardiovascular risk factors who presented with chest pain that was initially attributed to an ischemic nature; however, after a negative ischemic work-up, non-cardiac etiologies were considered after a thoracic mass was incidentally discovered during the non-cardiac imaging part of Lexiscan. Finally, the patient was diagnosed with multiple myeloma with metastasis to the lumbar spine. This case showed the importance of being cognizant of other causes of chest pain.
